# Supplementary material for: The interactome of intact mitochondria by cross-linking mass spectrometry provides evidence for coexisting respiratory supercomplexes
Source: Mol Cell Proteomics. 2017 Dec 8;17(2):216–32. doi: 10.1074/mcp.RA117.000470 (PMC5795388; doi:10.1074/mcp.RA117.000470)
Supplement: Supplemental Data [file supp_RA117.000470_133922_0_supp_23978_fzffwf.zip › spectra_annotation/mito_DR_spectra_annotation/200-1-4-1-6-1.pdf]

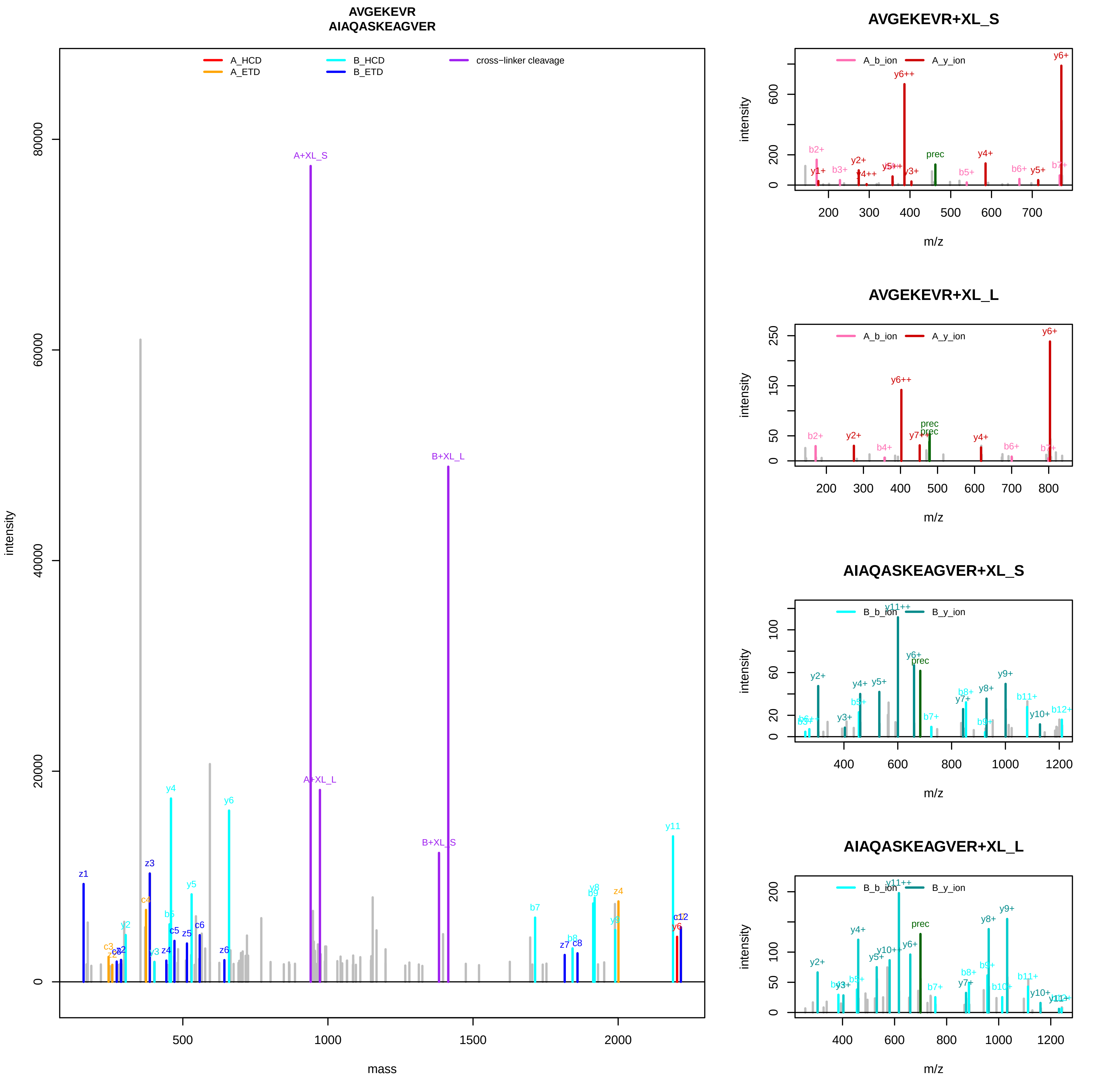
AVGEKEVR
AIAQASKEAGVER
A_HCD
A_ETD
B_HCD
B_ETD
cross−linker cleavage
intensity
mass
A+XL_S
B+XL_L
A+XL_L
B+XL_S
z1
z3
c3
c4
y2
y3
z4
y4
b5
c5
z5
y5
c6
z6
y6
b7
z7
b8
c8
y8
b9
y9
z4
y11
c12
y6
AVGEKEVR+XL_S
A_b_ion
A_y_ion
m/z
intensity
y6+
y6++
b2+
y2+
y1+
b3+
y4++
y5++
y3+
prec
y4+
b5+
b6+
y5+
b7+
AVGEKEVR+XL_L
y6+
y6++
prec
b2+
y2+
b4+
y7++
y4+
b6+
b7+
AIAQASKEAGVER+XL_S
B_b_ion
B_y_ion
y11++
y6+
prec
y2+
y4+
y5+
b5+
y3+
b8+
y7+
y8+
y9+
b11+
b7+
b9+
y10+
b12+
AIAQASKEAGVER+XL_L
y11++
y9+
y8+
prec
y4+
y6+
y10++
y5+
y2+
b8+
b9+
b11+
y7+
y3+
b5+
b7+
b10+
y10+
y11+
